# Crosstalk between the Warburg effect, redox regulation and autophagy induction in tumourigenesis

**DOI:** 10.1186/s11658-018-0088-y

**Published:** 2018-05-04

**Authors:** Mokgadi Violet Gwangwa, Anna Margaretha Joubert, Michelle Helen Visagie

**Affiliations:** Department of Physiology, Faculty of Health Sciences, University of Pretoria, Private Bag X323, Arcadia, 0007 South Africa

**Keywords:** Warburg effect, Autophagy, Oxidative stress, Cancer

## Abstract

Tumourigenic tissue uses modified metabolic signalling pathways in order to support hyperproliferation and survival. Cancer-associated aerobic glycolysis resulting in lactic acid production was described nearly 100 years ago. Furthermore, increased reactive oxygen species (ROS) and lactate quantities increase metabolic, survival and proliferation signalling, resulting in increased tumourigenesis. In order to maintain redox balance, the cell possesses innate antioxidant defence systems such as superoxide dismutase, catalase and glutathione. Several stimuli including cells deprived of nutrients or failure of antioxidant systems result in oxidative stress and cell death induction. Among the cell death machinery is autophagy, a compensatory mechanism whereby energy is produced from damaged and/or redundant organelles and proteins, which prevents the accumulation of waste products, thereby maintaining homeostasis. Furthermore, autophagy is maintained by several pathways including phosphoinositol 3 kinases, the mitogen-activated protein kinase family, hypoxia-inducible factor, avian myelocytomatosis viral oncogene homolog and protein kinase receptor-like endoplasmic reticulum kinase. The persistent potential of cancer metabolism, redox regulation and the crosstalk with autophagy in scientific investigation pertains to its ability to uncover essential aspects of tumourigenic transformation. This may result in clinical translational possibilities to exploit tumourigenic oxidative status and autophagy to advance our capabilities to diagnose, monitor and treat cancer.

## Background

**Fig. 1:**
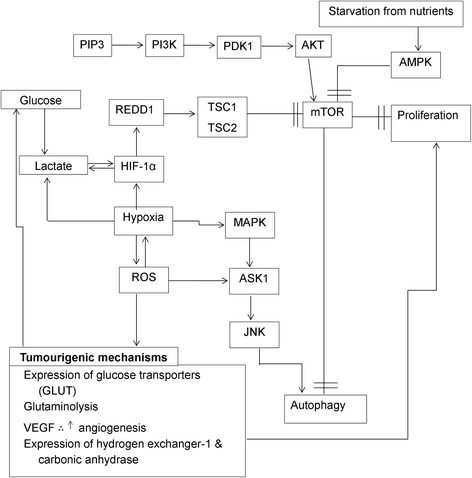
Benefits of tumourigenic metabolism. Tumourigenic metabolism is characterised by the Warburg effect with high ATP and lactate quantities produced in aerobic glycolysis. Furthermore, increased glycolysis and glutaminolysis are present in tumourigenic cells by means of increased expression of glucose and amino acid transporters resulting in increased production of ATP. Lactate production generates an acidic and hypoxic microenvironment which promotes tumourigenesis, invasion and metastasis. The hypoxic microenvironment results in autophagy induction via increased ROS production and subsequent JNK activation. Furthermore, hypoxia also induces expression of HIF-1α, which leads to inhibition of mTOR. In addition, PI3K/Akt signaling also results in inhibition of mTOR. The inhibition of mTOR results in subsequent autophagy induction. Images were created using Microsoft Word 2010 software

In non-tumourigenic cells, glycolysis is a highly regulated and conserved metabolic process in the cytoplasm in which glucose is converted to pyruvate by a series of enzymatic steps. Pyruvate is converted to acetyl coenzyme A, which produces adenosine triphosphate (ATP) via the tricarboxylic acid cycle in the mitochondria involving oxidative phosphorylation (OXPHOS) [1]. Glutaminolysis is a process whereby glutamine is converted to intermediates of the tricarboxylic acid cycle through a series of enzymatic steps. Initially, glutamine is oxidized, forming glutamate that is subsequently converted into α-ketoglutarate, which enters the tricarboxylic acid cycle to provide the metabolic intermediates [2]. However, tumourigenic cells possess aberrant metabolic programming that supports hyperproliferation, survival and long-term maintenance that is characterised by the Warburg effect and increased glycolysis and glutaminolysis (Fig. 1) [3, 4].

OXPHOS in the mitochondria generates 36 ATP molecules from 1 molecule of glucose compared to tumourigenic aberrant glycolysis that only produces 2 ATP molecules from 1 molecule of glucose. In order to compensate for the low ATP output per glucose molecule, tumourigenic cells increase the uptake of glucose by several ways including upregulated glucose transporter 1 (GLUT1) expression (Fig. 1) [5]. GLUT1 overexpression is found in several types of cancer and is associated with poor clinical outcomes in lung cancer, breast cancer, oesophageal cancer, hepatocellular carcinoma, gallbladder carcinoma, colorectal cancer, ovarian cancer and bladder cancer [6–8]. Lee et al. [9] reported that phosphorylation of GLUT1 on Ser226 by protein kinase C regulates glucose transport and GLUT1 deficiency syndrome also demonstrated impaired Ser226 phosphorylation. In addition, a known glucose uptake inducer, 12-O-tetradecanoyl-phorbol-13-acetate (TPA), increases GLUT1 cell surface localization and GLUT1 phosphorylation on Ser226 [9].

The increased requirement for energy production and synthesis of macromolecules results in increased transport of the required nutrients from the environment. Two key nutrients amongst these are glucose and glutamine. Tumourigenic cells consume glucose and glutamine at a higher rate when compared to non-tumourigenic or differentiated tissue and are therefore described as being addicted to glucose and glutamine [4, 10]. The increased consumption of glucose and glutamine yields upregulated carbon sources for anabolic processes and proliferation. The excess carbon is directed towards multiple pathways including glycolysis, glutaminolysis, fatty acid synthesis, nucleic acid production and the pentose phosphate pathway (PPP), resulting in synthesis of macromolecules including nucleotides, lipids and proteins [11].

The Warburg effect describes a hallmark of cancer where glycolysis results in lactate production even in aerobic conditions [1, 3, 4]. Furthermore, Warburg reported that tumourigenic cells exhibit defective mitochondrial OXPHOS that results in switching metabolic energy production to glycolysis. However, decades later we now have research indicating that the mitochondria in tumourigenic cells are not damaged or defective and OXPHOS still takes place in tumourigenic cells proportionally to oxygen supply. However, the rate of glycolysis is drastically upregulated, resulting in lactate production irrespective of oxygen availability [12]. Tumourigenic cells also metabolise glucose by means of the PPP, resulting in nicotinamide adenine dinucleotide phosphate hydrogen (NADPH) production. NADPH also promotes anabolism and favours tumourigenesis by enhancing the antioxidant defence system against hostile environments, radiation and chemotherapeutic compounds [13]. Furthermore, PPP increases generation of ribose-5-phosphate, which is required for the production of nucleic acids. In addition, NADPH also promotes fatty acid synthesis [14].

Increased glucose metabolism by means of tumourigenic glycolysis generates lactic acid and subsequently generates an acidic and hypoxic microenvironment which promotes tumourigenesis, invasion, metastasis and survival and is correlated with clinical prognosis and outcome. Hypoxia increases glucose consumption and subsequent glycolysis, re-enforcing the acidic and hypoxic microenvironment that is beneficial for tumourigenesis [15]. Acidic microenvironments are toxic to non-tumourigenic cells; however, in tumourigenic acidic microenvironments this promotes degradation of the extracellular matrix by proteinases, increases angiogenesis through vascular endothelial growth factor (VEGF) and inhibits the immune response to tumour antigens. As the extracellular matrix is degraded by the proteinases, the open space is invaded by tumourigenic cells. Further adaptations to promote acidic and hypoxic conditions include upregulation of hydrogen exchanger-1 and carbonic anhydrase [16].

Tumourigenesis is characterised by several genotypic and phenotypic metabolic alterations that allow for the increased metabolic activity and proliferation [17]. Cell metabolism is a complicated network of interconnected pathways and impacts overall tumour survival. Research is still ongoing in aberrant cancer metabolic processing and novel therapeutics may selectively target tumourigenic cells by obstructing the metabolic evolution. This is of great importance since the altered tumourigenic metabolism has been reported to be essential in limitless proliferation and resistance to apoptosis induction [18].

### Tumourigenic redox regulation

**Fig. 2:**
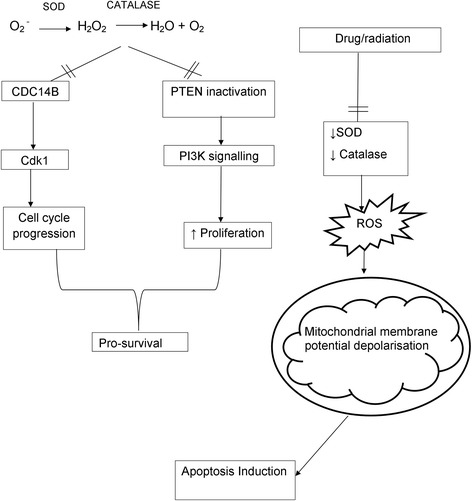
Tumourigenic ROS signalling. ROS inhibits PTEN activity that results in increased augmentation of PI3K/ Akt signalling and subsequently promotes proliferation. Furthermore, ROS promotes cell cycle progression by means of inhibiting phosphatase Cdc14B resulting in the activation of cyclin dependent kinase 1 (Cdk1). Furthermore, anti-tumourigenic compounds induce ROS production by means of inhibiting FOXM1 activity. This results in inhibition of enzymatic activity of antioxidant enzymes including SOD and catalase. The resulting increase ion ROS leads to the depolarisation of the mitochondrial membrane potential and apoptosis induction. Images were created using Microsoft Word 2010 software

A hallmark of cancer is increased production of reactive oxygen species (ROS) due to upregulated proliferation and glycolytic activity [19]. ROS are a group of molecules produced during metabolism and are fundamental signalling molecules at low concentrations. Production of mitochondrial ROS primarily occurs at the electron transport chain situated on the inner mitochondrial membrane during OXPHOS. ROS include the hydroxyl radical (OH), hydrogen peroxide (H_2_O_2_) and superoxide (O_2_^−^) [20]. Oxygen reduction by one electron produces superoxide, which is known as the precursor of most intracellular ROS. Superoxide is catalysed into hydrogen peroxide via an antioxidant defence mechanism in an effort to reduce danger elicited by ROS (Fig. 2) [21]. Subsequently, hydrogen peroxide is reduced to water and oxygen or partially reduced via transition elements to the hydroxyl radical [20, 21].

In addition to superoxide produced in mitochondrial metabolism, superoxide is also produced by NADPH oxidases that are located in the cellular membrane of various cell types including endothelial cells, neutrophils, eosinophils, monocytes and macrophages. Another source of superoxide generation is from the electron transport chain. The electron transport complexes include complex I (nicotinamide adenine dinucleotide hydrogen (NADH) dehydrogenase), complex II (succinate dehydrogenase), complex III (cytochrome *c* reductase), complex IV (cytochrome *c* oxidase) and complex V (mitochondrial F_1_F_0_ ATP synthase) [21–23]. Research indicates that the superoxide radical anion is produced by transfer of an unpaired electron from ubisemiquinone of complex I or complex III to an oxygen molecule [24].

The higher quantities of ROS produced in tumourigenic cells are beneficial for tumourigenesis and promote signalling pathways responsible for proliferation, aberrant metabolic activities and angiogenesis [25]. Hydrogen peroxide is well known for being membrane-permeable and is often utilised for intracellular signalling pertaining to proliferation and survival by means of reversible cysteine oxidation within proteins [25, 26]. Hydrogen peroxide oxidises cysteine residues on phosphatase and tensin homolog (PTEN), resulting in PTEN inactivation and subsequent enhancement of phosphoinositide 3-kinase (PI3K)/protein kinase B (Akt) signalling and promotes proliferation [19].

Upregulated ROS quantities in tumourigenic cells result in increased susceptibility to oxidative stress which causes damage to protein, lipids and deoxyribonucleic acid (DNA). The DNA damage is a double-edged sword since the DNA mutations potentially promote tumourigenesis and genomic instability or the cell undergoes cell death. Survival of tumourigenic cells is highly dependent on their capacity to regulate gene expression that controls innate antioxidant activity [27, 28]. Antioxidant defences include a superoxide dismutase (SOD) with manganese (Mn), which reduces superoxide that is created in the matrix. Superoxide quantities in the intermembrane space are regulated by copper and zinc SOD isozyme which is found in the cytoplasm. The intermembrane also contains cytochrome *c*, which is reduced by superoxide to produce oxygen. Subsequently, cytochrome *c* is still capable of transferring electrons to the terminal oxidase during the electron transport chain process [21]. Hydrogen peroxide can be detoxified by glutathione peroxidase, which is mainly found in the liver. Phospholipid-hydroperoxide glutathione is associated with detoxifying peroxides that are found in the membrane. Catalase, which is found in the peroxisomes, is known to detoxify hydrogen peroxide without cofactors, to water and oxygen. All these mechanisms are involved in maintaining a steady state between ROS and the antioxidant activity [29].

A cancer-specific isoform of pyruvate kinase, pyruvate kinase M2 (PKM2), confers an additional advantage to tumourigenic cells by providing them the advantage to withstand oxidative stress [30]. PKM2 is activated by fructose 1,6-bisphosphate and induces an active tetrameric confirmation. Phosphotyrosine-marked proteins activated by extracellular growth signals bind to PKM2 and convert PKM2 to a low activity confirmation by inducing expression of fructose 1,6-bisphosphate [31]. Furthermore, PKM2 promotes glycolysis and subsequent production of lactate in tumourigenic cells [32]. Modulation of PKM catalytic activity also regulates DNA and lipid synthesis required for cell proliferation and NADPH required for redox homeostasis [32].. Exposure to hydrogen peroxide or a thiol-oxidizing compound (diamide) induced oxidation of PKM2 at Cys358 in lung cancer (A549) cells. Furthermore, treatment with a reducing agent (dithiothreitol) restored PKM2 activity. Also, diamide induced impaired coimmunoprecipitation of endogenous PKM2 with flagged-tagged PKM2, and this was totally inhibited by exposure to a reducing agent (dithiothreitol) prior to immunoprecipitation. This suggests that oxidative stress induces PKM2 subunits to dissociate and results in less stable subunit association. Thus, PKM2 provides tumourigenic cells with an additional advantage to withstand oxidative stress. However, increasing PKM2 will compromise its pro-anabolic and antioxidant functions. Activation of PKM2 by small molecule compounds may be a potential future anti-cancer therapy to interfere with tumourigenic metabolism and antioxidant advantage [30]. These PKM2 activators bind to a pocket at the PKM2 subunit interface distinct from the binding site for fructose 1,6-bisphosphate and promote the association of PKM2 subunits into stable tetramers [33, 34]. Small molecule PKM2 activators mimic PKM1 in PKM2 expressing cells and interfere with anabolic metabolic processes including compromised production of ribose-phosphate and serine, which are essential precursors for generation of nucleotides, lipids and amino acids. Potential anticancer activity by small molecule PKM2 activators was confirmed when small molecule PKM2 activators abrogated the ability of tumourigenic cells to form cancer tumours in mice [33].

Nuclear factor kappa B (NF-kB), a redox-sensitive transcription factor, regulates the expression of several genes (cyclin D1, cyclin B1, interleukin (IL)-1, IL-2, granulocyte-macrophage colony stimulating factor (GM-CSF) and baculoviral IAP repeat-containing 3) that regulate inflammation, stress responses due to oxidative stress and apoptosis induction. Moreover, it has been suggested that NF-kB is a sensor for oxidative stress [35–37]. Low hydrogen peroxide quantities activate NF-kB and antioxidants inhibit NF-kB activation [26]. Reactive oxygen species activate NF-kB directly and additionally are involved in NF-kB activation by various other stimuli including tumour necrosis factor alpha (TNFα), phorbol ester, and IL-1 [37]. Oxidative stress resulting in TNFα activation leads to upregulation of antioxidant activity including SOD and catalase. However, NF-kB is essential for tumour progression since genes associated with NF-kB are frequently found in cancer. An NF-kB family member, *v-Rel* oncogene, has been shown to transform cells in vitro and in vitro [35].

Anticancer drugs that increase ROS production in cancer include bortezomib (Velcade), siomycin, thiostrepton and MG132, since these compounds inhibit the transcription activity of Forkhead Box M1 (FOXM1) [38]. FOXM1 is overexpressed in several types of cancer (prostate, lung, bladder, ovarian, colon, liver, breast stomach and pancreatic cancer) and reduces ROS by means of inducing expression of antioxidant enzymes including catalase, SOD2 and thioredoxin-dependent peroxidase reductase (PRDX3). Furthermore, exposure to FOXM1 inhibitors results in decreased tumour growth in breast and liver xenografts and sensitisation to cell death induced by DNA-damaging agents including doxorubicin and radiation [27, 39].

### Metabolic symbiosis between oxidative/aerobic tumour cells and hypoxic/glycolytic cells

Monocarboxylate transporters (MCTs) of the *SLC16A* gene family determine substrate availability, the metabolic path of lactate and acidity balance within the tumour and the microenvironment [40]. MCT-1, MCT-2, MCT-3 and MCT-4 are essential for proton-linked transportation, and previous studies have shown that MCT-1, MCT-2 and MCT-4 are upregulated in several types of cancer including breast, ovary, colon, liver and lung cancer [41]. MCT-4 is upregulated by hypoxia and contributes to acidification of the microenvironment by means of glycolysis that produces lactate. MCT-1 is upregulated by oxygen and MCT-1 expression is repressed by hypoxia. Furthermore, MCT-1 promotes the transport of lactate into the cell and subsequent utilization of lactate to produce energy [42]. Research further demonstrated that inhibition of MCT-1 did not affect cell survival in cells propagated in growth medium containing glucose; however, cell death induction was prominent when MCT-1 was silenced in cells propagated in growth medium not containing glucose. Inhibition of MCT-1 in mouse models injected with Lewis lung carcinoma cells or colon colorectal tumourigenic cells (WiDr) resulted in decreased tumour growth [43]. Cells expressing MCT-4 thus promote uptake of glucose and subsequent utilization via glycolysis to produce lactate. MCT-1-expressing cells import lactate and utilize it as a substrate in the tricarboxylic acid and as a consequence exhibit stem cell-like characteristics. Cells expressing MCT-1 are rich in mitochondria and produce high quantities of metabolic intermediates and ATP [44, 45]. Inhibition of MCT-1 can thus be a potential therapeutic option since silencing of MCT-1 abrogates the metabolic symbiosis between oxidative/aerobic tumour cells and hypoxic/glycolytic cells [44].

### Autophagy

Autophagy is another crucial defence mechanism utilised by the cells in response to oxidative stress [39]. Autophagy (derived from Greek and meaning “self-eating”) is a compensatory mechanism that produces energy from cytoplasmic contents including damaged and/or redundant organelles and proteins which subsequently prevent the accumulation of waste products, thereby maintaining homeostasis [20].

Autophagy of non-selective cytosolic contents by lysosomes is classified into 3 categories, namely macroautophagy, microautophagy and chaperone-mediated autophagy. Microautophagy involves cytosolic contents being taken up by lysosomes directly through an invagination of the lysosomal membrane. In chaperone-mediated autophagy, proteins are translocated across the lysosomal membrane accompanied with chaperone proteins (including heat shock protein 70) that are recognised by the lysosomal membrane receptor, lysosomal-associated membrane protein 2A (LAMP-2A), leading to their unfolding and degradation [46]. In macroautophagy (hereafter referred to as autophagy), a part of the cytoplasm is taken up by a membrane sac called the isolation membrane (also known as the phagophore). The isolation membrane continues to expand and engulfs the aggregates by creating double membrane vesicles called autophagosomes. The latter matures and fuses with lysosomes, creating autolysosomes [47, 48]. Autophagy is generally a non-selective degradation process. However, in rare cases, autophagy is selective of damaged or excess organelles. Selective autophagy is characterised depending on the contents of the degraded cargo: mitophagy (mitochondria), pexophagy (peroxisomes), reticulophagy (endoplasmic reticulum), ribophagy (ribosomes), lipophagy (lipid droplets), aggrephagy (protein aggregates), nucleophagy (nuclear contents) and xenophagy (invasive microbes) [48].

Autophagy serves in an adaptive capacity, promoting cell survival during periods of cellular stress including nutrient starvation, hypoxia, ATP/adenosine monophosphate (AMP) ratio alteration and intracellular ROS. However, certain conditions may result in pro-death autophagy induction [20, 49]. The mechanisms in which autophagy switches from pro-survival to pro-death is not well understood yet, but the involvement of Beclin 1 is indicated [50]. Beclin 1 is a protein associated with autophagy and is essential for embryonic development and autophagy initiation in non-tumourigenic cells and, if present, will distinguish whether the fate of the cell is pro-death or pro-survival [51]. Autophagy is frequently used as a pro-survival mechanism in stressful conditions and contributes to the turnover of cellular contents at a basal level, whereas starvation-induced autophagy provides amino acids for the gluconeogenesis and protein synthesis that is essential in nutrient-deprived conditions [48, 50, 52]. In other pro-survival adaption roles, autophagy contributes to chemotherapy and radiotherapy resistance. Radiation therapy, chemotherapy (doxorubicin, temozolomide and etoposide), histone deacetylase inhibitors, rapamycin, imatinib and anti-hormonal therapy (tamoxifen) induce pro-survival autophagy in human cancer cell lines. If autophagy is inhibited, the efficacy of these therapies increases [49, 50].

### Molecular pathways involved in autophagy

Autophagy is a process that requires multiple signalling aspects creating a network of regulatory complexes and protein-protein interactions which aid in a fully functional process [53] (Fig. 1). The process of autophagy can be broken down into several steps, namely induction, vesicle nucleation, expansion and maturation, breakdown and recycling [54]. Autophagy is induced by the unc-51 like autophagy activating kinase (ULK1) complex that consists of the ULK1/ULK2 protein, focal adhesion kinase family interacting protein 200 kDa (FIP 200), scaffold and the Hop/Rev7/Mad2 (HORMA) domain-containing proteins autophagy-related protein (ATG) 13 and ATG101 [55]. Subsequently, the ULK1 complex regulates the formation of the vacuolar protein sorting 34 (VPS34) complex consisting of VPS34 protein, VPS15 protein, Beclin 1 and ATG14 [55, 56]. The phagophore formation in response to stress is dependent on VSP 34 ultraviolet irradiation resistance-associated gene (UVRAG), B-cell lymphoma-2 associated X protein-interacting factor 1 (BIF 1), ATG14-like protein (ATG14L) and RUN domain and cysteine-rich domain containing Beclin 1-interacting protein (Rubicon) [46, 57].

### Mammalian target of rapamycin

Autophagy induction is regulated by a serine/threonine protein kinase, mammalian target of rapamycin (mTOR) (Fig. 1). The mTOR signalling pathway is integrated by mammalian target of rapamycin complex 1&2 (mTORC1/mTORC2) [50]. mTORC1 consists of mTOR, regulatory-associated protein mTOR complex 1 (Raptor), and mammalian lethal with SEC13 protein 8 (mLST8) and regulates protein synthesis, proliferation, autophagy, metabolism and the stress response [58, 59]. Raptor facilitates recruitment of the substrate to mTORC1 by means of binding to the TOR signalling (TOS) motif. mTORC1 also contains proline-rich AKT substrate 40 kDa (PRAS40) and DEP domain-containing mTOR-interacting protein (Deptor) [45]. mTORC2 consists of mTOR, mLST8, rapamycin-insensitive companion of mTOR complex 2 (Rictor), mammalian stress-activated map kinase interacting protein (mSIN1), and protein observed with Rictor (Protor1/2) and controls cell survival and polarity [58–60].

Both mTORC complexes are stimulated by growth complexes; however, only mTORC1 is activated by amino acids. Glutaminolysis generates α-ketoglutarate, which induces the translocation of mTORC1 to the lysosome and also activates mTORC1, as demonstrated by increased ribosomal protein S6 kinase (S6K) and S6 phosphorylation [61]. This subsequently leads to inhibition of ULK1, which results in autophagy inhibition. However, a decrease in nutrients or oxygen results in mTORC1 inhibition by means of ULK1 phosphorylation and subsequently autophagy is induced and an autophagosome is formed [11]. The elongation of this structure is dependent on the ATG 12&5 aided by ATG 7&10. ATG 12&5 interact with ATG 16, which then forms a complex and is subsequently added onto the phospholipids in the membrane, in a process that is similar to ubiquitination and SUMOylation. ATG 4 protease aids the previously mentioned reaction and continues to cleave microtubule-associated protein 1A/1B-light chain 3 (LC3), which is further incorporated into the membrane subsequently closing the double membrane structure. The latter fuses with the lysosome and is further degraded, leading to autolysosome formation [25, 57, 62].

Amino acids are the major stimuli for mTORC activation, but the specific mechanistic profile remains elusive; for example, the specific amino acids that regulate mTORC1 are still unclear. Studies have indicated that mTOR senses amino acids (leucine, glutamine and arginine) indirectly within the lysosomal lumen, which requires Rag guanine triphosphatases (GTPases). Activated Rag GTPases recruit mTORC to the lysosome, where Rheb GTPase activates mTORC1 [63]. There are four Rag proteins of interest, namely RagA, RagB, RagC and RagD. RagA and RagB form a heterodimer with RagC and RagD, respectively. RagA/RagB complexes bind directly to mTORC1, and overexpression of constitutively active RagA/RagB protein renders mTORC1 insensitive to amino acid deprivation [64]. However, mTORC is active in fibroblasts lacking RagA and RagB (RagA/B knockout (KO)). Leucine and arginine stimulate mTORC1 activation. However, arginine and leucine failed to induce activation of mTORC in RagA/B KO fibroblasts. Glutamine stimulated mTORC1 irrespective of Rag /B expression (control and RagA/B KO fibroblasts) at the lysosome, indicating that glutamine stimulation of mTORC1 is independent of Rag GTPases [63].

### Class I phosphoinositol 3 kinase pathway

The phosphoinositol 3 kinases (PI3K), a family of lipid enzymes, are crucial in autophagy, proliferation, differentiation, motility, intracellular trafficking and cell survival. PI3K phosphorylates the 3′-hydroxyl position of the inositol ring in phosphatidylinositides including phosphatidylinositol (PI) and phosphatidylinositol-4,5-bisphosphate (PIP2) [65]. PIP2 phosphorylation results in the formation of phosphatidylinositol-3,4,5-triphosphate, which activates protein kinase B (Akt) [50]. PIP3 binds to Akt and subsequently Akt moves to the cell membrane, where Akt is activated by PDK1 and mTORC2 phosphorylation by Thr308 and Ser473, respectively [66]. Akt inhibits tuberous sclerosis complex (TSC) by phosphorylating TSC2 at Ser939 and binding to 14–3-3 protein (Fig. 1). Subsequently TSC promotes activation of RAS homolog enriched in brain (Rheb) and mTOR stimulation inhibition and inhibits autophagy induction [66]. Furthermore, activation of autophagy by means of rapamycin increases phosphorylation of Akt, which provides negative feedback and inhibits autophagy induction [67].

Deregulation of PI3K/Akt/mTOR signalling is associated with high-grade tumours (grade 3–grade 4) and tumourigenesis [68]. Thus, modulation of the PI3K/Akt pathway would modify autophagy inhibition or induction and could be a target for chemotherapy [69]. Compounds that target PI3K are currently being investigated for anticancer activity by means of autophagy induction include NVP-BEZ235 (dactolisib), XL-765, PX-866, NVP-BKM120 (buparlisib), GDC-0941 (pictilisib), XL-147, NVP-BYL719, GSK-2636771 and GDC-0032 (taselisib) [43]. NVP-BEZ235 (dactolisib), a dual inhibitor of PI3K and mTOR, exerts dose-dependent antiproliferative activity, inhibits Akt phosphorylation (Ser473) and induces apoptosis in human glioblastoma cells (U87). However, in vivo studies utilizing nude rats and NOD/SCID mice in orthotopic xenograft models of glioblastoma demonstrated that NVP-BEZ235 (dactolisib) exerted no survival benefit or inhibition of tumour growth [70]. NVP-BKM120 (buparlisib) targets PI3K by inhibiting the catalytic subunit p110α of PI3K by competitive binding of the lipid kinase domain on the ATP binding site [71]. NVP-BKM120 (buparlisib) possesses antiproliferative and pro-apoptotic activity in several types of tumourigenic cell lines including breast, glioblastoma, osteosarcoma, ovary and prostate and is to date enlisted in more than 80 clinical trials. Thus, targeting the PI3K/Akt/mTOR pathway for increased effective chemotherapy remains promising, but requires more research [71, 72].

### Mitogen-activated protein kinase family

Mitogen-activated protein kinase (MAPK) signalling is essential in proliferation, migration, differentiation, apoptosis induction, autophagy induction and sensitivity to chemotherapy. The MAPK family consists of c-Jun N-terminal kinase or stress-activated protein kinase (JNK or SAPK), extracellular signal-regulated kinase (ERK1/2), big MAP kinase 1 (Erk1/2) and p38 and is involved in cell proliferation and survival processes [73–75]. The ERK pathway is activated by growth factors, cytokines, ultraviolet irradiation, genotoxic agents, oxidative stress and hormones. This results in the phosphorylation of cytoplasmic signalling proteins including G alpha interacting protein, which abolishes the inhibitory protein (Gi3) of autophagy. ERK also targets nuclear components such as ternary complex factor (TCF) transcription factors which are essential for inducing expression of c-Fos and myelocytomatosis oncogene (*c*-Myc), subsequently promoting cell survival proliferation and motility [73, 74].

Various stressful conditions including oxidative stress activate JNK and p38 pathway signalling by means of apoptosis signal-regulated kinase 1 (ASK1) in several types of cancer including human colon cancer cells (HCT116) (Fig. 1) [76]. This is due to ROS oxidizing two cysteine residues in the redox centre located on thioredoxin. This induces formation of a disulphide bond between Cys32 and Cys35, leading to thioredoxin dissociating from ASK1 and oligomerisation of ASK1. Furthermore, ROS activates JNK by inhibiting JNK-inactivating phosphatases by means of oxidation of a catalytic site, cysteine to sulfenic acid. This results in promotion of JNK activation [75]. Thus, activation and inhibition of phosphatases by ROS are essential for cell signalling pertaining to JNK and p38 [73–75].

JNK, another member of the MAPK signalling family, comprises 3 isoforms of which the third isoform influences autophagy by phosphorylating B-cell lymphoma 2 (Bcl-2), which has proapoptotic functions. This results in a disturbance of the bond between Beclin 1 and Bcl-2, thereby inducing autophagy [62, 77]. Beclin 1 is regulated by JNK by phosphorylating c-Jun (transcription factor), which regulates Beclin 1 expression. Furthermore, JNK stimulate ATG5 to induce autophagy in which ATG expression is influenced by transcriptional factors including Forkhead Box 3 (FOXO3). AMPK aids in the accumulation of nuclear FOXO3; thus activating it which will eventually lead to the induction of autophagy [50].

The RAS family is the regulator of the MAPK/ERK pathway, and mutations in the RAS family are associated with cancer even under sufficient nutrient supply [74]. Human cancer cell lines presenting with *H-ras*- or *K-ras*-activating mutations demonstrate higher levels of autophagy induction and increased ROS levels. Inhibition of autophagy correlates with decreased cell growth, suggesting that autophagy is essential for tumour cell survival, and that suppression of autophagy in *Ras*-driven cancers may be an effective treatment [47].

### 5′-adenosine monophosphate-activated protein kinase

5′-Adenosine monophosphate-activated protein kinase (AMPK), a heterotrimeric protein complex, is an essential stress and energy sensor [78]. AMPK consists of alpha (catalytic), beta and gamma (both regulatory) subunits and is activated by numerous kinases through phosphorylation and the increase of calcium in the cytosol through calmodulin-dependent protein kinase kinase beta (CAMKK-ß). AMPK senses energy by binding directly to AMP, ATP or ADP, resulting in conformational changes of the enzyme and activation, thus promoting phosphorylation and subsequently preventing further dephosphorylation of the energy molecules [79].

Oxidative stress and starvation lead to AMPK activation, resulting in mTOR inhibition and subsequently autophagy induction accompanied characterised by autophagosome formation [80]. Isoquinoline alkaloids including liensinine, isoliensinine, dauricine, cepharanthine and hernandezine directly activate AMPK and induce autophagy in several cancer cell lines including a cervical cancer cell line (HeLa), alveolar adenocarcinoma cell line (A549), breast adenocarcinoma cell line (MCF-7), prostate cancer cell line (PC3), liver cancer cell lines (HepG2 and Hep3B) and human non-small cell lung carcinoma cell line (H1299) [81].

Studies have suggested that low DNA damage results in pro-survival autophagy induction. However, high levels of DNA damage result in pro-death autophagy induction via activation of ATM, activation of AMPK and repression of mTORC1 [82]. The DNA damage response (DDR) is associated with increased ROS generation and initiates ATM signalling that activates AMPK and subsequent autophagy induction. Furthermore, ionizing radiation activates ATM, which in turn activates tumour suppressor tuberin (TSC2) by means of serine/threonine-protein kinase 11 (STK11) and the AMPK metabolic pathway, resulting in inhibition of mTORC1, and subsequently activates ATG1 [83].

Tumourigenic cells are autophagy-addicted since tumourigenic cells tend to induce autophagy constitutively by means of metabolic reprogramming and AMPK activation. AMPK regulates the autophagy-dependent amino acid recycling system with FIP200 and ULK1 [84–86]. Even under nutrient-sufficient conditions, increased mTOR activity inhibits ULK1 activation by ULK1 phosphorylation on Ser757, thereby abrogating the interaction between ULK1 and AMPK [87].

AMPK is also responsible for the turnover of mitochondria via mitophagy. ULK 1 and ULK2 form stable associations with AMPK in which AMPK phosphorylates and activates ULK1, triggering mitophagy [88]. In cells where the endogenous ULK was replaced with AMPK (and where the phosphorylation sites were defective and replaced by alanine) damaged mitochondrial morphology and reduced mitochondrial membrane potential after nutrient deprivation were present [89].

### Hypoxia-inducible factor

Tumourigenic cells depend on autophagy induction in order to survive hypoxia and lower ROS production [90]. Hypoxia-inducible factor (HIF) is a transcriptional regulator during hypoxia that constitutively expresses the beta subunit and an oxygen regulated alpha subunit, which is degraded under normal conditions (oxygen-rich) [91]. Hypoxia results in decreased ubiquitination of the alpha subunit, thereby providing stability. The alpha subunit binds to hypoxia responsive element sequences, thus facilitating the metabolic shift from OXPHOS to glycolysis. In oxidative stressful environments or nutrient-deprived conditions, HIF-1 is capable of stimulating AMPK and subsequently induces autophagy. Furthermore, HIF-1 stimulates Regulated in development and DNA damage response 1 (REDD1) in hypoxia. REDD1 activates the TSC1/2 complex, thereby halting mTOR and activating autophagy [57].

HIF-1 also activates transcription of the gene encoding the BH3 domain protein BNIP3, which induces mitochondrial autophagy (mitophagy) by means of competing with Beclin-1 for binding privileges with Bcl-2. Therefore Beclin-1 is free to induce autophagy. BNIP3-induced autophagy was originally suggested to be hypoxia-specific, but studies conducted in HIF-1α-null mouse embryo fibroblasts demonstrated that mitochondrial autophagy was essential for cell survival in prolonged hypoxic conditions [92].

### Cancer stem cells

The significant intra-tumour heterogeneity in tumour tissue plays an essential role in resistance to conventional anticancer treatments including chemotherapy and radiation [93]. Tumour heterogeneity refers to heterogeneous cellular populations with a hierarchical organization governed by cancer stem cells also known as progenitor-like cells or stem cell-like cells [94, 95]. Furthermore, cancer recurrence and subsequent metastasis are largely due to disseminated tumour cells which are cancer stem cells that survived the initial treatment, generally referred to as minimal residual disease. The presence of these disseminated tumour cells in the bone marrow and lymph nodes is associated with poor prognosis and high mortality rates [96]. In addition, cancer stem cells are the cause of all tumourigenic cells contained within the tumour and are capable of forming genetic clones of tumourigenic cells contained within the malignant tissue [97, 98]. Cancer stem cells possess high tumourigenic activity and stem cell-like abilities including capabilities of self-renewal, multi-potent differentiation and multiple DNA repair mechanisms, making them cell death resistant [99]. Cancer stem cells renew themselves by remaining in an undifferentiated state. Cancer stem cells also express specific surface markers, including CD44, CD133 and enzyme aldehyde dehydrogenase (ALDH) [100].

Presently, there is no consensus on the metabolic characteristics of cancer stem cells; however, numerous studies suggest glycolytic dependency instead of mitochondrial respiration. Research confirms that cancer stem cells are more glycolytic than other differentiated cancer cells in vitro and in vivo [101]. However, there are also studies that support cancer stem cell proliferation that utilizes mitochondrial respiration. Moreover, cancer stem cells’ mitochondria possess increased mass and mitochondrial membrane potential, which is a reflection of mitochondrial function, higher mitochondrial ROS production and higher oxygen consumption compared to differentiated cancer cells that generate their energy mainly via glycolysis [102–105]. Cancer stem cells adapt metabolic- and signalling pathways contingent to the microenvironmental changes by shifting energy production from one pathway to the other [106]. Differentiation or a hypoxic microenvironment results in cancer stem cells from several tumour types switching from oxidative to glycolytic metabolism in order to compensate for deficient mitochondrial machinery [107].

Studies indicate that intracellular ROS levels are significantly lower when compared to non-tumourigenic stem cells, which may be due to upregulated expression of members of the free radical scavengers system [108]. ROS have been implicated in metastasis via cell migration, invasion and angiogenesis. One of the proposed signalling mechanisms involves CD13 negatively regulating ROS with a resultant increase of stem cell-like abilities and increased ROS scavenger capacity in human liver cancer stem cells [109]. Furthermore, hydrogen peroxide activates canonical wingless-type mouse mammary tumour virus (MMTV) integration site family (Wnt) signalling, which is vital for malignancy regulation and tumourigenesis [110, 111].

Cancer stem cells have a superior antioxidant defence system that includes a CD44 variant isoform containing variable exon 8–10 (CD44v8–10) and cysteine/glutamate antiporter (xCT). Both have been implicated to be significant when considering resistance to anticancer treatment [112, 113]. CD44v8–10 interacts with cysteine transporter xCT (SLC7A11), resulting in stabilization of cysteine transporter xCT, thereby possibly providing protection against redox stress due to ROS generation. The cysteine transporter xCT acts as a sodium-independent transporter that regulates the exchange of extracellular cysteine for intracellular glutamate and supports reduced glutathione (GSH) synthesis, which acts as an intracellular buffer. CD44v-xCT-GSH is associated with resistance to anticancer agents. Cysteine availability is rate-limiting to GSH synthesis, and the activity of xCT is therefore crucial to resistance to oxidant-dependent anticancer agents [114]. Ectopic expression of CD44v8–10 was induced after ROS-enhancing chemotherapy in a patient with a hereditary cancer predisposition syndrome commonly associated with p53 mutations (Li-Fraumeni syndrome) [115]. Thus, the CD44v-xCT-GSH axis is of importance when considering resistance to a microenvironment rich in oxidative stress and subsequent anticancer treatments dependent on ROS production [110, 113, 115].

Oxidative stress activates the Wnt pathway and promotes expression of CD44 and *c*-Myc. Furthermore, high quantities of hydrogen peroxide activate the canonical beta-catenin/Wnt signalling pathway by means of the thioredoxin-like protein nucleoredoxin (NRX) [116, 117]. Furthermore, CD44v8–10 and *c*-Myc have an inversed expression pattern in vitro and in vivo. The latter negative correlation is especially prominent at the invasive front of the tumour enriched with cancer stem cells [110]. Another study verified the existence of crosstalk between ROS and the Wnt pathway. Wnt signalling induced ROS production mediated by NADPH oxidase 1 (Nox1). This occurred by increased levels of the Ras-related C3 botulinum toxin substrate 1 (Rac1)-GTP through activation of Rac1, guanine nucleotide exchange factors (GEFs) and vav guanine nucleotide exchange factor 2 (Vav2) by proto-oncogene tyrosine-protein kinase Src-dependent tyrosine phosphorylation [118]. Moreover, Nox1-generated ROS inactivate NRX through oxidation and disrupt the NRX-dishevelled (Dvl) complexes, thereby activating the Wnt-β-catenin pathway involved in proliferation of both healthy and malignant cells [118].

A γ-secretase-mediated intracellular domain of CD44 promotes aggressive glioma growth and a stem cell-like phenotype by means of CREB-binding protein (CBP)/p300-dependent enhancement of HIF-2α activity. Furthermore, expression of CD44 correlates with hypoxia-induced gene signatures and poor survival rates in human glioblastoma multiforme patients [119]. A hypoxic bone marrow microenvironment is a stem- and progenitor cell niche that acts as a sanctuary for cancer stem cells. HIF-1α expression is upregulated in the hypoxic bone marrow niche. HIF-1α induces expression of stromal cell-derived factor 1 (also known as C-X-C motif chemokine 12 (CXCL12)), resulting in an increase in adhesion, migration and homing of circulating C-X-C chemokine receptor type 4 (CXCR4)-positive progenitor cells into the ischemic tissue [120]. This suggests that a hypoxic bone marrow microenvironment represents a conditional stem and progenitor cell niche in which HIF-1α-induced stabilization and activation of CXCL12 and CXCR4 facilitate recruitment and retention of progenitor cells. In this context, HIF-1α may represent an important molecular target within the tumour microenvironment [121].

Recent research indicates that autophagy promotes the stem cell-like characteristics present in cancer stem cells in several types of cancer including breast cancer, pancreatic cancer, colon cancer, hepatocarcinoma, osteosarcoma and bladder cancer [122, 123]. Inhibition of autophagy resulted in a decrease in stem cell surface markers (POU Class 5 Homeobox 1 Transcript Variant OCT4B4, SRY-Box 2, Homeobox Transcription Factor Nanog and CD44) in breast cancer cell lines (MCF-7 and MDA-MB-468) [123, 124]. Furthermore, autophagy inhibition in osteosarcoma cancer stem cells decreased cancer cell surface markers and enhanced sensitivity to hypoxic conditions and exposure to cisplatin, resulting in increased cell death induction [122–124]. Thus, identification of mechanisms underlying cancer stem cell characteristics and the development of novel approaches to target them holds promise for the therapeutic elimination of cancer stem cells and the complete eradication of tumours [120–124].

## Conclusion

Research has shown that autophagy mediates cancer cell metabolism, allowing for hyperproliferation by providing amino acids and other building blocks when the macromolecules are unavailable from other sources [125]. Autophagy has also been shown to be required for tumourigenic cell survival during hypoxic stress in order to combat oxidative stress [90]. Hypoxia, which is a characteristic of cancerous cells, produces moderately high levels of ROS leading to the induction of autophagy [126]. ROS enhances the PI3K pathway, which is already hyperactivated in cancerous cells. It is stated in the literature that activation of the PI3K pathway, which is hyperactivated by cell growth producing growth factor signalling, leads to increased proliferation and cellular mobility and promotes cell survival. ROS also activate HIF, which is a pathway activated upon hypoxic conditions and as is known to be a prominent state in tumour cells [77]. Tumourigenic cells activate HIF-1, resulting in transcription activation, in order to adapt to the hypoxic condition. The transcription targets of the HIFs promote cell survival in hypoxic conditions, shifting metabolism in order to increase aerobic glycolysis and angiogenesis [127, 128].

Hypoxia and nutrient deprivation increase the AMP/ATP ratio, which subsequently leads to the activation of AMPK, thereby inhibiting mTORC1 and activating autophagy [62]. Furthermore, ROS (hydrogen peroxide) is also known to induce autophagy in cancer by means of oxidising ATG4, followed by the removal of lipids and protein maturation of ATG4, resulting in LC3-associated autophagosomes [125]. Increased H_2_O_2_ production causes oxidative stress and AMPK phosphorylation by an upstream kinase (AMPKK), resulting in autophagy induction [62].

Autophagy and oxidative stress are both involved in a complex signalling network in tumourigenesis pertaining to major processes including proliferation, metabolism, angiogenesis, oxidative stress and resistance to cell death (autophagy and apoptosis) and oxidative stress [129]. In addition, tumourigenic cells possess increased metabolic activities which produce high ROS quantities contributing to hyperproliferation and increased cell survival, in turn contributing to chemo- and radiosensitivity [130, 131]. The interactions of the pathways and processes are evidently tightly linked, but are not well documented in the literature as one complete or linked process. The manner in which metabolism autophagy as well as ROS interacts along with the pathways involved will contribute to novel and more effective methods to target tumourigenic cells in order to reduce the high cancer prevalence. The bimodal nature of ROS and its influence on autophagy by means of pro-survival and pro-death signalling requires elucidation in order to identify novel biochemical targets for chemotherapy and increased efficacy in current treatment options.

## Abbreviations

ALDH: Aldehyde dehydrogenase
AMP: Adenosine monophosphate
AMPK: 5′-Adenosine monophosphate-activated protein kinase
ASK1: Apoptosis signal-regulated kinase 1
ATG: Autophagy-related protein
ATG14L: ATG14-like protein
ATP: Adenosine triphosphate
Bcl-2: B-cell lymphoma 2
BIF 1: B-cell lymphoma-2 associated X protein-interacting factor 1
CAMKK-ß: Calmodulin-dependent protein kinase kinase beta
CBP: CREB-binding protein
CD44v8-10: CD44 variant isoform containing variable exon 8-10
Cdk1: Cyclin dependent kinase 1
CXCL12: C-X-C motif chemokine 12
CXCR4: C-X-C chemokine receptor type 4
DDR: DNA damage response
Deptor: DEP domain-containing mTOR-interacting protein
DNA: Deoxyribonucleic acid
Dvl: Dishevelled
ERK1/2: Extracellular signal-regulated kinase
FIP 200: Focal adhesion kinase family interacting protein 200 kDa
FOXM1: Forkhead Box M1
FOXO3: Forkhead box 3
GLUT1: Glucose transporter 1
JNK: C-Jun N-terminal kinase
TPA: 12-O-Tetradecanoyl-phorbol-13-acetate
xCT: Cysteine/glutamate antiporter
GSH: Glutathione
GM-CSF: Granulocyte-macrophage colony stimulating factor
GTPases: Guanine triphosphatases
HIF: Hypoxia-inducible factor
H_2_O_2_: Hydrogen peroxide
OH: Hydroxyl radical
LAMP-2A: Lysosomal-associated membrane protein 2A
Mn: Manganese
mLST8: Mammalian lethal with SEC13 protein 8
mSIN1: Mammalian stress-activated map kinase interacting protein
mTOR: Mammalian target of rapamycin
LC3: Microtubule-associated protein 1A/1B-light chain 3
MAPK: Mitogen-activated protein kinase
MCTs: Monocarboxylate transporters
MMTV: Mouse mammary tumour virus
*c*-Myc: Myelocytomatosis oncogene
Nox1: NADPH oxidase 1
NADH: Nicotinamide adenine dinucleotide hydrogen
NADPH: Nicotinamide adenine dinucleotide phosphate hydrogen
NF-kB: Nuclear factor kappa B
OXPHOS: Oxidative phosphorylation
PPP: Pentose phosphate pathway
PTEN: Phosphatase and tensin homolog
PI: Phosphatidylinositol
PIP2: Phosphatidylinositol-4,5-bisphosphate
PI3K: Phosphoinositide 3-kinase
PRAS40: Proline-rich AKT substrate 40 kDa
Protor: Protein observed with Rictor
Akt: Protein kinase B
PKM2: Pyruvate kinase M2
Rac1: Ras-related C3 botulinum toxin substrate 1
S6K: Ribosomal protein S6 kinase
Rictor: Rapamycin-insensitive companion of mTOR complex 2
Raptor: Regulatory-associated protein mTOR complex 1
REDD1: Regulated in development and DNA damage response 1
Rheb: RAS homolog enriched in brain
ROS: Reactive oxygen species
Rubicon: RUN domain protein as Beclin 1 interacting and cysteine-rich containing
HORMA: Scaffold and the Hop/Rev7/Mad2
STK11: Serine/threonine-protein kinase 11
SAPK: Stress-activated protein kinase
O_2_: Superoxide
SOD: Superoxide dismutase
PRDX3: Thioredoxin-dependent peroxidase reductase
NRX: Thioredoxin-like protein nucleoredoxin
TCF: Ternary complex factor
TOS: TOR signalling
TSC: Tuberous sclerosis complex
TNFα: Tumour necrosis factor alpha
TSC2: Tumour suppressor tuberin
UVRAG: Ultraviolet irradiation resistance-associated gene
ULK1: Unc-51 like autophagy activating kinase
VPS34: Vacuolar protein sorting 34
VEGF: Vascular endothelial growth factor
Vav2: Vav guanine nucleotide exchange factor 2
Wnt: Wingless-type MMTV integration site family
